# Cognitive dysfunction in naturally occurring canine idiopathic epilepsy

**DOI:** 10.1371/journal.pone.0192182

**Published:** 2018-02-08

**Authors:** Rowena M. A. Packer, Paul D. McGreevy, Hannah E. Salvin, Michael J. Valenzuela, Chloe M. Chaplin, Holger A. Volk

**Affiliations:** 1 Department of Clinical Science and Services, Royal Veterinary College, Hatfield, Hertfordshire, United Kingdom; 2 Sydney School of Veterinary Science, Faculty of Science, The University of Sydney, New South Wales, Australia; 3 Brain and Mind Centre, Sydney Medical School, The University of Sydney, Sydney, New South Wales, Australia; Nathan S Kline Institute, UNITED STATES

## Abstract

Globally, epilepsy is a common serious brain disorder. In addition to seizure activity, epilepsy is associated with cognitive impairments including static cognitive impairments present at onset, progressive seizure-induced impairments and co-morbid dementia. Epilepsy occurs naturally in domestic dogs but its impact on canine cognition has yet to be studied, despite canine cognitive dysfunction (CCD) recognised as a spontaneous model of dementia. Here we use data from a psychometrically validated tool, the canine cognitive dysfunction rating (CCDR) scale, to compare cognitive dysfunction in dogs diagnosed with idiopathic epilepsy (IE) with controls while accounting for age. An online cross-sectional study resulted in a sample of 4051 dogs, of which n = 286 had been diagnosed with IE. Four factors were significantly associated with a diagnosis of CCD (above the diagnostic cut-off of CCDR ≥50): (i) epilepsy diagnosis: dogs with epilepsy were at higher risk; (ii) age: older dogs were at higher risk; (iii) weight: lighter dogs (kg) were at higher risk; (iv) training history: dogs with more exposure to training activities were at lower risk. Impairments in memory were most common in dogs with IE, but progression of impairments was not observed compared to controls. A significant interaction between epilepsy and age was identified, with IE dogs exhibiting a higher risk of CCD at a young age, while control dogs followed the expected pattern of low-risk throughout middle age, with risk increasing exponentially in geriatric years. Within the IE sub-population, dogs with a history of cluster seizures and high seizure frequency had higher CCDR scores. The age of onset, nature and progression of cognitive impairment in the current IE dogs appear divergent from those classically seen in CCD. Longitudinal monitoring of cognitive function from seizure onset is required to further characterise these impairments.

## Introduction

In human neurology it is widely recognised that epilepsy is associated with cognitive impairment, with impaired cognition considered so prevalent that there are calls for repeated cognitive screening for all epilepsy patients [1]. Cognitive impairments in epilepsy vary in severity, onset and progression. Impairments range from mild learning difficulties through to severe mental decline [2] and may be static or progressive, transient or permanent. Indeed, cognitive profiles in epilepsy are considered to be as heterogeneous as the epileptic syndromes themselves [3]. The cause of epilepsy and the neuropathology associated with it often dictate the expression of cognitive comorbidities [4]. Greater severity of cognitive impairment is seen in symptomatic/structural epilepsies (where seizures are a symptom of an underlying problem, such as stroke, trauma, infection, congenital brain malformation, tumours, or metabolic disorders [5]), than in idiopathic epilepsy (IE) [6], where epilepsy is the direct result of a presumptive but unknown genetic defect(s) [5]. Even still, cognitive dysfunction is common in IE, a cluster of syndromes that represents approximately 20% of all epilepsies [7].

There is debate as to whether cognitive impairments in epilepsy are (i) progressive, and as a result of the recurrent seizure activity that characterises epilepsy, (ii) static, and present from the onset of epilepsy, or (iii) due to other co-morbid neurological diseases. Part of this early debate was further complicated by studies suggesting a slow cognitive decline with increased duration of epilepsy, but epilepsy duration is heavily confounded by ageing that is itself associated with cognitive decline. It is possible that the nature and progression of decline varies between individuals with epilepsy, and multiple processes that compromise cognition may occur contemporaneously.

Epilepsy is characterised by recurrent seizure activity, and in some epilepsy syndromes, evidence suggests that recurrent seizure activity has a role in cognitive decline. A review of twenty longitudinal studies that examined cognitive outcomes in patients with epilepsy concluded that there is a ‘mild but definite’ relationship between seizures and mental decline [8]. Seizures in the human temporal lobe (TLE) transiently impair cognition and steadily damage hippocampal circuitry, leading to progressive memory loss [9]. Patients with a longer duration of TLE have a lower IQ than those with a shorter duration [10] and, in a longitudinal study of TLE patients, progressive decline in cognition preferentially occurred in patients with ongoing seizures. In contrast, patients who became seizure-free after resection of the dysfunctional hippocampal structures improved or recovered from their previous deficits [11].

Other studies suggest that cognitive deficits in chronic epilepsy cannot be interpreted exclusively as long-term effects of recurrent seizure activity, and that these early deficits are better categorised as initial symptoms of epilepsy, the severity of which may then increase in the long-term course of the disease or take the course of physiological ageing [3]. Indeed, approximately half of newly diagnosed children or adults with epilepsy have demonstrable cognitive or behavioural difficulties on testing [12–14]. Contrary to previous assumptions, age regressions for healthy controls and patients with TLE appear to run in parallel, with TLE patients performing worse than controls, while the distance between groups remained stable over time [15]. This may indicate that the cognitive problems in epilepsy evolves around the time of epilepsy onset or perhaps even earlier. It is possible that epilepsy interferes with the developmental trajectory of brain networks underlying cognition [16], and that the underlying aetiology of epilepsy is involved in the mechanism of permanent cognitive deficits [17]. Even where the rate of cognitive decline in epilepsy is slow, patients who start at a lower baseline cognitive level than healthy individuals may reach a clinically significant threshold of impairment earlier in life [15].

Finally, it is possible that cognitive declines in adult and elderly patients with epilepsy are not solely a consequence of the seizure disorder. Evidence of co-morbidity between epilepsy and dementia is mounting. Two population-based investigations of medical comorbidities demonstrated a significantly higher rate of dementia and Alzheimer’s disease (AD) in patients with epilepsy compared with controls. [18, 19]. In the EURODEM project, eight case–control studies were reanalysed and the risk for AD was found to be significantly raised in patients with established epilepsy (duration >10 years) [20]. A 8-year longitudinal study of 4505 epilepsy patients aged 50–75 years, compared with age-matched controls with other medical disorders, revealed an age-accelerated risk of dementia [21]. A parallel relationship has also been found, with epilepsy also being more likely in dementia and AD patients; indeed, in one study, the incidence ratio of epilepsy in individuals diagnosed with AD was elevated nearly 87-fold [22]. This may indicate a bidirectional relationship between the disorders, such that dementia may precede or follow the development of epilepsy [20].

Given these disease patterns in humans it is interesting that idiopathic epilepsy is the most common chronic neurological condition in domestic dogs, with prevalence estimates from 0.6% [23] - 0.75% [24]. Age of onset of IE is most commonly between 6 months and 6 years [25]. Although there is increasing evidence of neurobehavioural comorbidity in the dog, including anxiety and ADHD-like behaviour [26–28], the effect of IE on canine cognition has yet to be explored. Canine cognitive dysfunction (CCD) or ‘canine dementia’ is a recognised neurobehavioural syndrome in aged dogs, characterized by deficits in learning, memory and spatial awareness, as well as changes to social interactions and sleeping patterns [29]. CCD is common in aged dogs [30–32], but underdiagnosed [33] with a large community-based sample estimating it affects ~12% of dogs greater than 10 years and doubling in prevalence every 2 years thereafter [34]. In addition, anxiety has been observed in around half of CCD cases, as also seen in Alzheimer’s disease in humans [35]. With the life expectancy of companion animals increasing as a consequence of improvements in veterinary care and nutrition, geriatric disorders are becoming increasingly important in veterinary medicine [36]. Moreover, since both CCD and IE having the potential to compromise quality of life [34, 37], co-morbidity of these disorders may have a major negative impact on canine welfare. The primary aim of this study was therefore to investigate whether dogs with IE are at higher risk of CCD when accounting for age. If cognitive impairments were found in the IE population, the secondary aims were:

1. To characterise the age of onset, nature and progression of any cognitive impairment observed in the population of dogs with IE;
2. To identify disease and treatment-related risk factors for CCD within the population of dogs with IE; and

Finally, this study aimed to identify other environmental and physical risk factors for CCD in the overall population.

## Methods

### Study design and recruitment

A cross-sectional study was conducted using an online survey platform (SurveyMonkey; www.surveymonkey.co.uk) between June and September 2016, to explore the impact of IE upon CCD. To avoid response bias associated with revealing the central research question, the survey was entitled the ‘Mature Dog Study’, with the stated aim being to explore the impact of canine dogs' health and training upon their behaviour (See S1 File for full survey). Consent was gained via a statement in the opening page detailing the storage and use of respondents’ data in accordance with the Data Protection Act 1998. The survey was promoted primarily via social media, breed clubs and veterinary practices. The study was approved by the local ethics committee (RVC Animal Welfare and Ethics Committee; approval number 2016/U301).

### Subjects

The study aimed to recruit both dogs affected by IE, and controls with no history of recurrent seizure activity. Dogs were screened for IE by consecutive diagnostic questions, beginning with whether they had ever had a seizure (yes/no). Those owners answering no were categorised into the control group. For those answering yes, three further screening questions were posed: (i) whether their dog had 2 or more seizures that were at least 24 hours apart; (ii) whether their dog’s first seizure occurred between the ages of 6 months and 6 years; and (iii) whether their vet had carried out blood and urine tests on their dog and found no identifiable cause for their dog’s seizures. If the owner answered yes to all three questions, the dog was considered to meet the tier I diagnostic criteria for the International Veterinary Epilepsy Task Force (IVETF) [38] and thus was classified as affected. Dogs who had experienced seizure activity, but whose owners only responded ‘yes’ to one or two of the above screening questions, were excluded from the study.

CCD is considered a geriatric onset condition, where prevalence increases exponentially with age. Previous studies have focused on dogs over 8 years of age (e.g. the Senior Dog Survey [34]); however, this age limit may exclude dogs with early-onset CCD. Therefore, a lower age limit of 3 years was set as an inclusion criterion for this study, with no upper age limit. set This allowed for a broader range of dogs that could reveal how cognitive dysfunction may develop over time in an adult population.

### Behavioural data

Cognitive impairment was measured using the canine cognitive dysfunction rating scale (CCDR), a psychometrically validated tool that quantifies the frequency and progression of thirteen behaviours which, when abnormal, concur with veterinary diagnoses of canine dementia almost 80% of the time [34]. The CCDR focuses on problems related to memory (lack of recognition of owners, house-soiling), orientation (staring blankly, getting lost in the home), apathy (reduced time spent active, avoiding contact with owners), impaired olfaction (difficulty finding food) and locomotion. Questions are included in Table 1, with dogs receiving an overall score out of 80. The diagnostic threshold for canine cognitive dysfunction is set at a summed CCDR score of ≥50.

**Table 1:** Canine cognitive dysfunction rating (CCDR) scale from salvin et al. (2011).

Question	(1) Never	(2) Once a month	(3) Once a week	(4) Once a day	(5) >Once a day	Score
How often does your dog pace up and down, walk in circles and/or wander with no direction or purpose?						
How often does your dog stare blankly at the walls or floor?						
How often does your dog get stuck behind objects and is unable to get around?						
How often does your dog fail to recognise familiar people or pets?						
How often does your dog walk into walls or doors?						
How often does your dog walk away while, or avoid, being patted?						
	**Never**	**1–30% of times**	**31–60% of times**	**61–99% of times**	**Always**	**Score**
How often does your dog have difficulty finding food dropped on the floor?						
	**Much less**	**Slightly less**	**The same**	**Slightly more**	**Much more**	**Score**
Compared with 6 months ago, does your dog now pace up and down walk in circles and/or wander with no direction or purpose						
Compared with 6 months ago does your dog now stare blankly at the walls or floor						
Compared with 6 months ago does your dog urinate or defecate in an area it has previously kept clean (if your dog has never house-soiled tick ‘the same’)						
Compared with 6 months ago does your dog have difficulty finding food dropped on the floor						x 2
Compare with 6 months ago does your dog fail to recognise familiar people or pets						x 3
	**Much more**	**Slightly more**	**The same**	**Slightly less**	**Much less**	**Score**
Compared with 6 months ago is the amount of time your dog spends active						

To account for the effect of environmental factors upon cognition, a history of previous training was taken using questions from a recent study of common training activities [39]. For early life training, owners were asked to report puppy class attendance in the following categories: 0 (never attended), 1–10, 11–20, >20. For adult training, owners were asked to report attendance of six activities: obedience classes, agility, gundog training, ringcraft, conformational showing and flyball. For each category, owners responded with their dog’s current or previous attendance and were allocated a score from 0–2 (0 = No: Never attended, 1 = Yes: Attended but has now stopped, 2 = Yes: Still attends), and an overall adult training score out of 12 was calculated.

Finally, owners were asked to report what training aids and methods they used to train their dog. Thirteen aids/methods were considered punishment-based (bark-activated citronella collar, prong collar, Pet Corrector^TM^, bark collar, water pistol, verbal punishment (e.g. shouting or telling off), electric collar, choke chain, electronic boundary fence, physical punishment e.g. smacking when the dog does something wrong, Husher^TM^, shutting away when the dog behaves badly, pulling back on the lead when the dog pulls) and five were considered reward-based (food reward, stroking or patting, verbal praise, playing and clicker training). Whether the owner used solely punishment-based methods, solely reward-based methods, or a mix of both methods was recorded.

### Clinical data

All owners reported their dog’s breed, sex, neuter status, age (years) and weight (kg). For the sub-population of dogs with IE, the following additional clinical data were collected: age at first seizure, seizure frequency in the past three months, and experience of cluster seizures: more than one seizure in a 24-hour period; and status epilepticus: a seizure lasting >5 minutes or two seizures with incomplete recovery of consciousness inter-ictally [38]. Owners were also asked to report their dog’s current anti-epileptic drug (AED) treatment and historic response to AEDs.

### Statistical analysis

Descriptive statistics were performed in SPSS Statistics v 23 (SPSS, Inc., Chicago) and modelling and graphing in R 3.3.3 [40]. Two outcome variables were considered:

1. Overall CCDR score out of 80;
2. CCDR diagnosis as a binary variable based on the optimal diagnostic cut-off (< or ≥50).

We considered the effects on these two outcome variables of the following potential predictors: age (years), sex, neuter status, bodyweight (kg) and idiopathic epilepsy diagnosis. To determine these variables’ effect upon CCD, linear and binary logistic regression modelling were used to evaluate potential predictors separately. Results of the univariate analyses were used to determine which factors to include in the subsequent multivariate models, with those liberally associated (p<0.2) being carried forward, to ensure that all potentially predictive variables were studied [41]. Biologically plausible interactions between variables were tested. Generalised linear models were used to model the effect of IE on CCDR and CCD (1/0). Age (years) was included as a variable in all models. To improve model fit, polynomials for continuous variables were examined. Model fit was checked by visually inspecting the distribution, and the homogeneity of variance, of the residuals, and the Akaike information criterion (AIC) values of each iteration. The intercept is stated for all multivariate models, the expected mean value of the response variable (Y) when all predictors (X) equal 0.

Within the IE group, the following predictors of interest were considered for their effect on CCD: age at first seizure, time since first seizure (epilepsy duration), report of cluster seizures and/or status epilepticus, current AED treatment, number of AEDs used and historic drug response. To characterise specific differences in CCD between control and IE cases, differences in the scores of the thirteen CCDR sub-questions of were compared using linear regression, controlling for age in all models. All tests were two-sided and P <0.05 was considered to be significant. Data are presented as mean ± standard deviation (SD), or median (25^th^-75^th^ quartile), where appropriate.

## Results

### Study population

Responses were received from the owners of 4710 dogs, of which 4051 were eligible for inclusion. The remainder were excluded due to a history of seizures without meeting diagnostic criteria for a Tier I level diagnosis, or due to being incomplete. Almost three quarters of the sample were purebred (71.4%), over half of which were registered with a kennel club in their country (66.7%, n = 1923). There were 173 different breeds represented, the top three being the Border Collie (9.2%, n = 371), Cavalier King Charles Spaniel (6.5%, n = 265) and Labrador Retriever (6.1%, n = 248). Most dogs in the sample were neutered, with 42.1% (n = 1707) being neutered females and 38.3% (n = 1553) being neutered males, 12.2% (n = 494) being entire males, and 7.3% (n = 297) being entire females. The median age of the overall population was 8.17 years (5.58–10.75), and the median age of the control population was 8.30 years (5.75–10.92).

A total of 286 dogs met the minimum three criteria required for IVETF tier I diagnostic certainty for IE. Of those dogs meeting tier I, 17% also achieved a atier II certainty diagnosis, as they had unremarkable fasting and post-prandial bile acids, magnetic resonance imaging of the brain and cerebrospinal fluid analysis [38]. Dogs with IE (n = 286) were significantly younger and heavier than controls (p<0.001). Dogs with IE were more likely to be male and neutered than controls (p<0.001). Dogs with IE had a significantly lower adult training score (epilepsy: 0 (0–1); control: 1 (0–2), p<0.001), but there was no difference in the training method used (% reward only: IE: 31.4%, control: 29.3%, p = 0.450).

### Prevalence of cognitive dysfunction

Using the CCDR diagnostic cut-off value of ≥50, 63 dogs (1.6%) in the overall population,were considered CCD + and 3988 (98.4%) CCD-. CCD was most common in the oldest age group (>14 years; 14.2%). In the epilepsy group, the majority of CCD+ cases were aged 4.1–6.0 years old (Table 2).

**Table 2:** Prevalence of CCD across different age groups, stratified by clinical group.

Age category	Overall (n = 4051)			Epilepsy only (n = 286)			Control only (n = 3765)		
	N	CCD +	CCD -	N	CCD +	CCD -	N	CCD +	CCD -
<4.0 years	439	0.5%	99.5%	48	4.2%	95.8%	391	0.0%	100.0%
4.1–6.0years	752	1.1%	98.9%	91	6.6%	93.4%	661	0.3%	99.7%
6.1–8.0years	805	0.7%	99.3%	76	2.6%	97.4%	729	0.5%	99.5%
8.1–10.0 years	809	0.1%	99.9%	44	0.0%	100.0%	765	0.1%	99.9%
10.1–12.0 years	607	1.0%	99.0%	19	5.3%	94.7%	588	0.9%	99.1%
12.1–14.0 years	428	2.3%	97.7%	6	0.0%	100.0%	422	2.4%	97.6%
>14.0 years	211	14.2%	85.8%	2	0.0%	100.0%	209	14.4%	85.6%

### Risk factors for CCD

Relevant signalment, disease and environmental variables were screened for their association with CCD at the univariate level. Five variables were found to differ between the CCD + and CCD–groups in univariate analyses. Compared with CCD- dogs, those classified as CCD+ were found to be significantly older, more likely to have epilepsy, be male, lighter, and have encountered fewer training activities (Table 3).

**Table 3:** Univariate associations between the presence of CCD (CCDR ≥50) and signalment, clinical and lifestyle variables.

Variable	Category	CCD -	CCD +	OR	95% CI	p
Age (years)	Continuous	-	-	1.41	1.26–1.57	**<0.001**
Bodyweight (kg)	Continuous	-	-	0.95	0.92–0.97	**<0.001**
Sex	Female	98.9%	1.1%	0.58	0.35–0.98	**0.040**
	Male	98.0%	2.0%	1 (base)		
Neuter status	Neutered	98.3%	1.7%	0.51	0.23–1.13	0.095
	Entire	99.1%	0.9%	1 (base)		
Idiopathic epilepsy	Affected	92.6%	3.8%	2.86	1.47–5.54	**0.002**
	Control	98.6%	1.4%	1 (base)		
Training method	Mixed	98.4%	1.6%	-0.138	0.87–1.98	0.622
	Reward-only	98.6%	1.4%	1 (base)		
Training history	Continuous	-	-	0.38	0.23–0.53	**<0.001**

**Fig 1:**
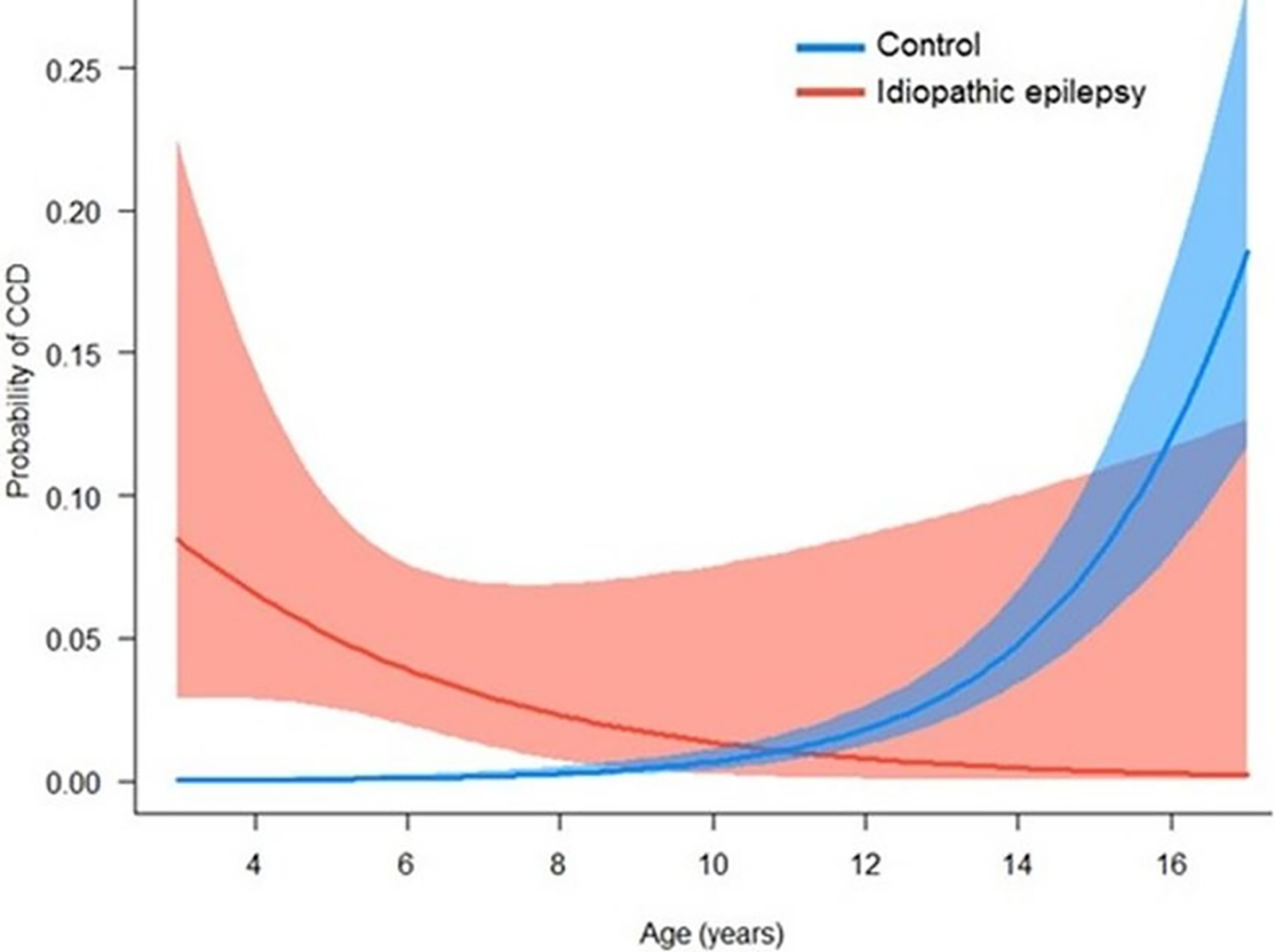
Probability of being affected by canine cognitive dysfunction (CCD) by age in dogs with epilepsy (n = 286) and controls (n = 3765). Results based on a generalised linear model with CCD as the binary outcome variable.

**Fig 2:**
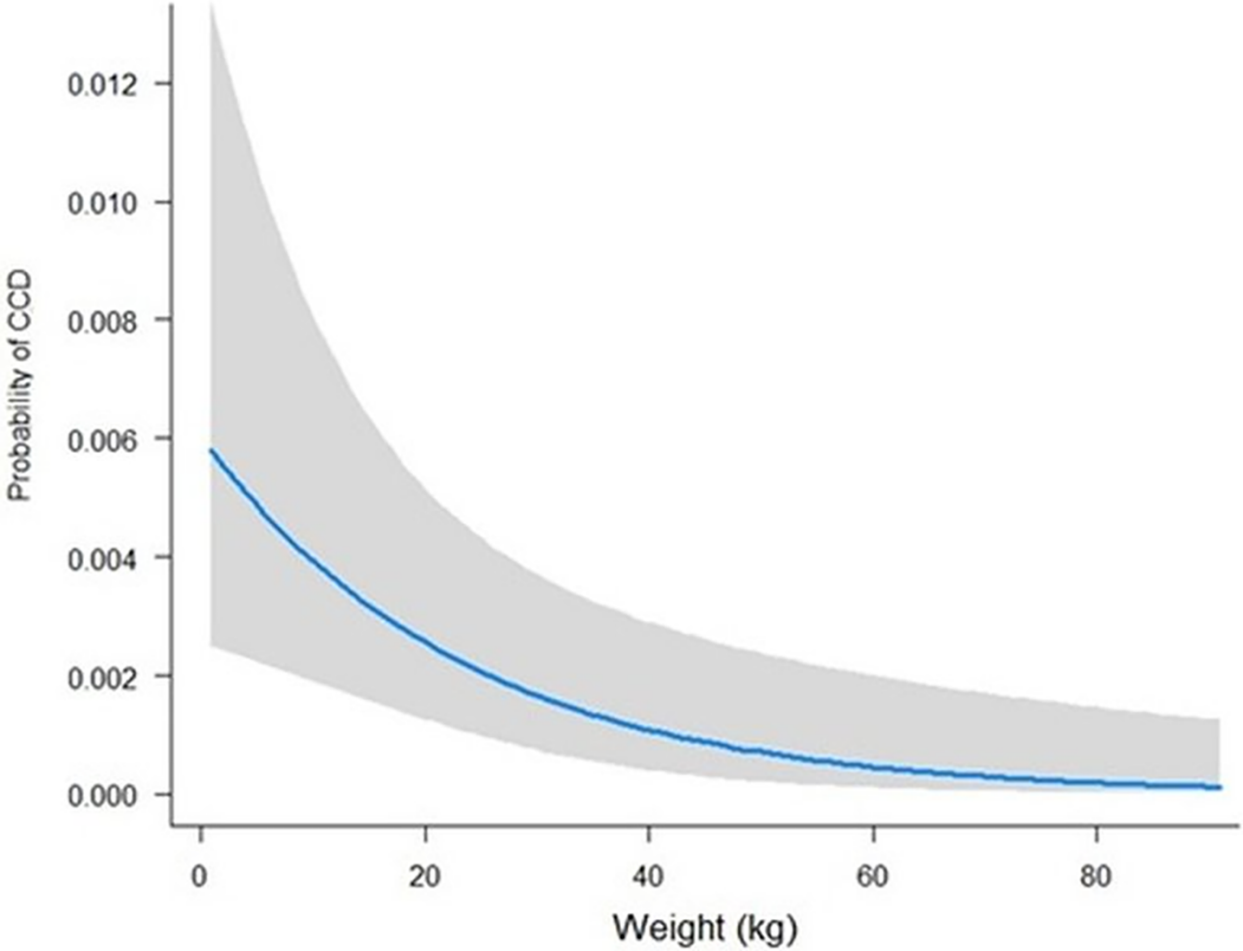
Probability of being affected by canine cognitive dysfunction (CCD) across weight (kg) in the control population. N.B. Age is kept constant (8 years old) to examine the effect of weight in isolation.

Variables liberally associated with CCD at the univariate level were taken forward into binary logistic regression modelling. In the final multivariate model, four variables remained significant: age, IE, bodyweight and training history (Table 4), with a significant interaction between age and IE diagnosis. The risk of CCD in healthy dogs increased exponentially with age beyond 10 years; however, risk of CCD in dogs with epilepsy was elevated at a comparatively young age (< 4 years) (Fig 1). When controlling for age, light dogs were at an elevated risk of CCD than heavier dogs (Fig 2).

**Table 4:** Multivariate model of risk factors for CCD in a population of 4051 dogs.

Variable	Sub-category	Coefficient	SE	Z	p
Intercept	-	-0.44	1.14	-0.38	0.702
Age (years)	-	-0.27	0.19	1.46	0.144
Idiopathic epilepsy	Affected	8.47	1.35	6.27	**<0.001**
	Control	*Reference*			
Bodyweight (kg)	-	-0.04	0.02	-2.81	**0.005**
Training history	-	-0.26	0.13	-2.00	**0.045**
Age (years) * Idiopathic epilepsy	-	0.77	0.20	-3.96	**<0.001**

N.B. “*” indicates an interaction term between two variables

### Risk of cognitive dysfunction in dogs under 8 years

In the study population aged >96 months (8 years), dogs with IE were underrepresented relative to the population aged ≤96 months, possibly due to the significantly reduced lifespan associated with epilepsy in dogs (≤96 months: 10.78% IE cases (215/1996); >96 months: 3.46% IE cases (71/2055)). To refine the association between epilepsy and CCD in the middle-aged canine population, binary logistic regression models were repeated including only dogs ≤96 months. Two effects remained significant: IE and the interaction beween IE and age (Table 5). In this model, age (years), weight (kg) and training history were no longer significant predictors of CCD diagnosis in this age range; however, age was retained in the final model due to its inclusion in a significant interaction term with IE.

**Table 5:** Multivariate model of risk factors for CCD in dogs ≤96 months of age (n = 1996 dogs).

Variable	Sub-category	Coefficient	SE	z	p
Intercept	-	-1.93	1.30	-1.49	0.137
Age (years)	-	-0.21	0.25	-0.84	0.399
Idiopathic epilepsy	Affected	7.57	2.68	2.83	**0.005**
	Control	*Reference*			
Age (years) * Idiopathic epilepsy	-	0.83	0.42	-1.95	**0.050**

N.B. “*” indicates an interaction term between two variables

**Fig 3:**
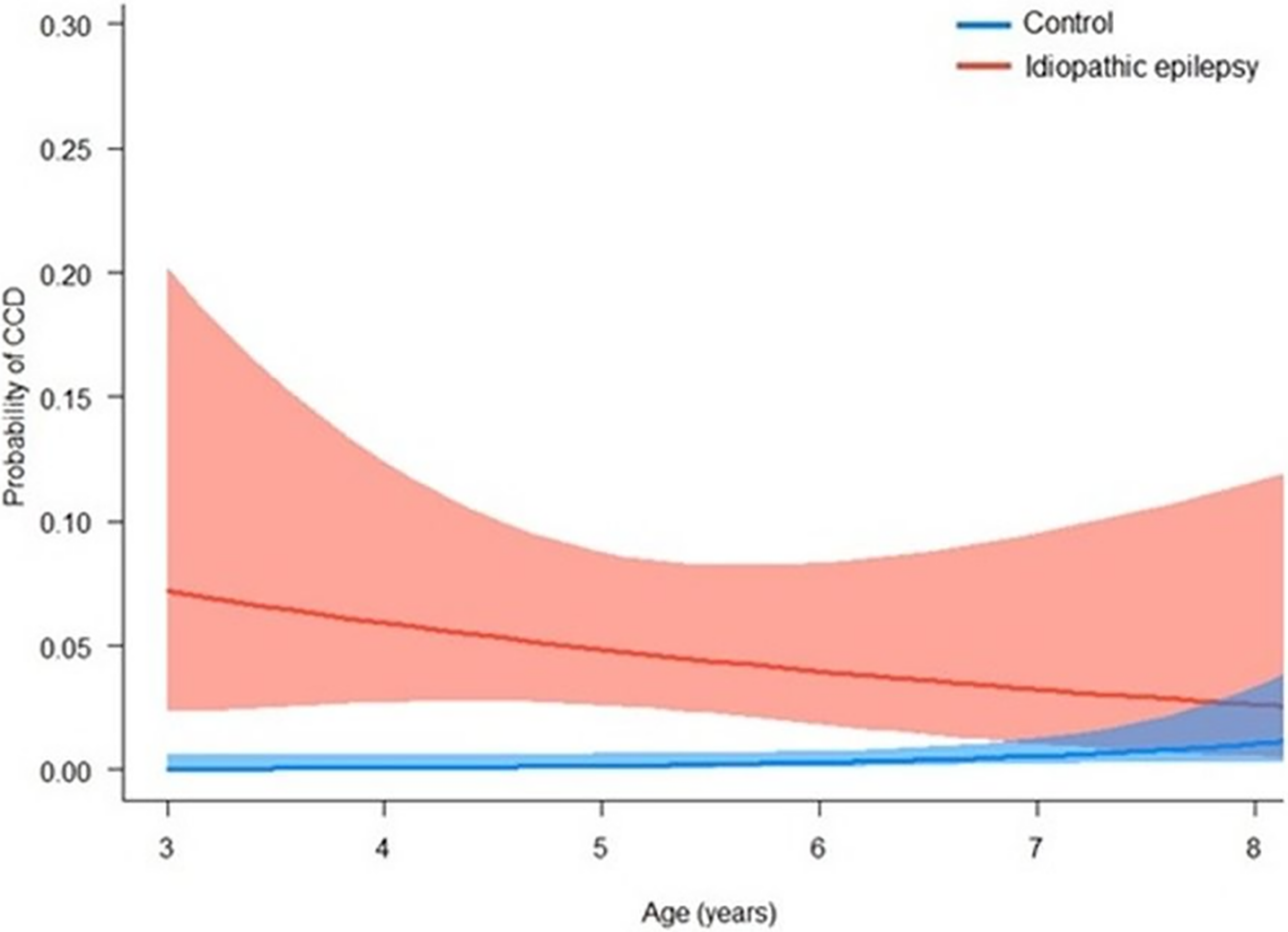
Probability of being affected by canine cognitive dysfunction (CCD) by age in dogs with epilepsy (n = 286) and controls (n = 3765). Data are based on a generalised linear model with CCD as the binary outcome variable.

In this sub-population, dogs with epilepsy were at a significantly elevated risk of CCD than controls until around 7 years of age. The risk of CCD in controls remained extremely low (probability <0.05) for the entire age range (Fig 3).

### CCDR score

The CCDR is a continuous score from 16–80. To model risk factors associated with an elevated CCDR score rather than for CCD+/- as a binary trait, linear regression modelling was used. When CCDR was modelled as a continuous variable all variables from the binary model remained significant, with the exception of training history (p>0.05). In addition, age was significant when fitted as a third degree polynomial, which improved model fit compared to first or first and second degree polynomials alone (Table 6).

**Table 6:** Generalised linear model of risk factors for CCDR score in a population of 4051 dogs.

Variable	Sub-category	Estimate	SE	t	p
Intercept	-	36.17	1.71	22.48	**<0.001**
Age (years)	Continuous	0.74	0.49	1.50	0.133
Age (years) ^ 2 (quadratic)	Continuous	-0.18	0.05	-3.39	**<0.001**
Age (years) ^ 3 (cubic)	Continuous	0.01	0.01	4.78	**<0.001**
Idiopathic epilepsy	Affected	4.497	1.095	4.11	**<0.001**
	Control	*Reference*			
Bodyweight (kg)	Continuous	-0.02	0.01	-2.39	**0.017**
Age (years) * Idiopathic epilepsy	Continuous	0.47	0.16	2.98	**0.003**

N.B. * represents an interaction between two variables

**Fig 4:**
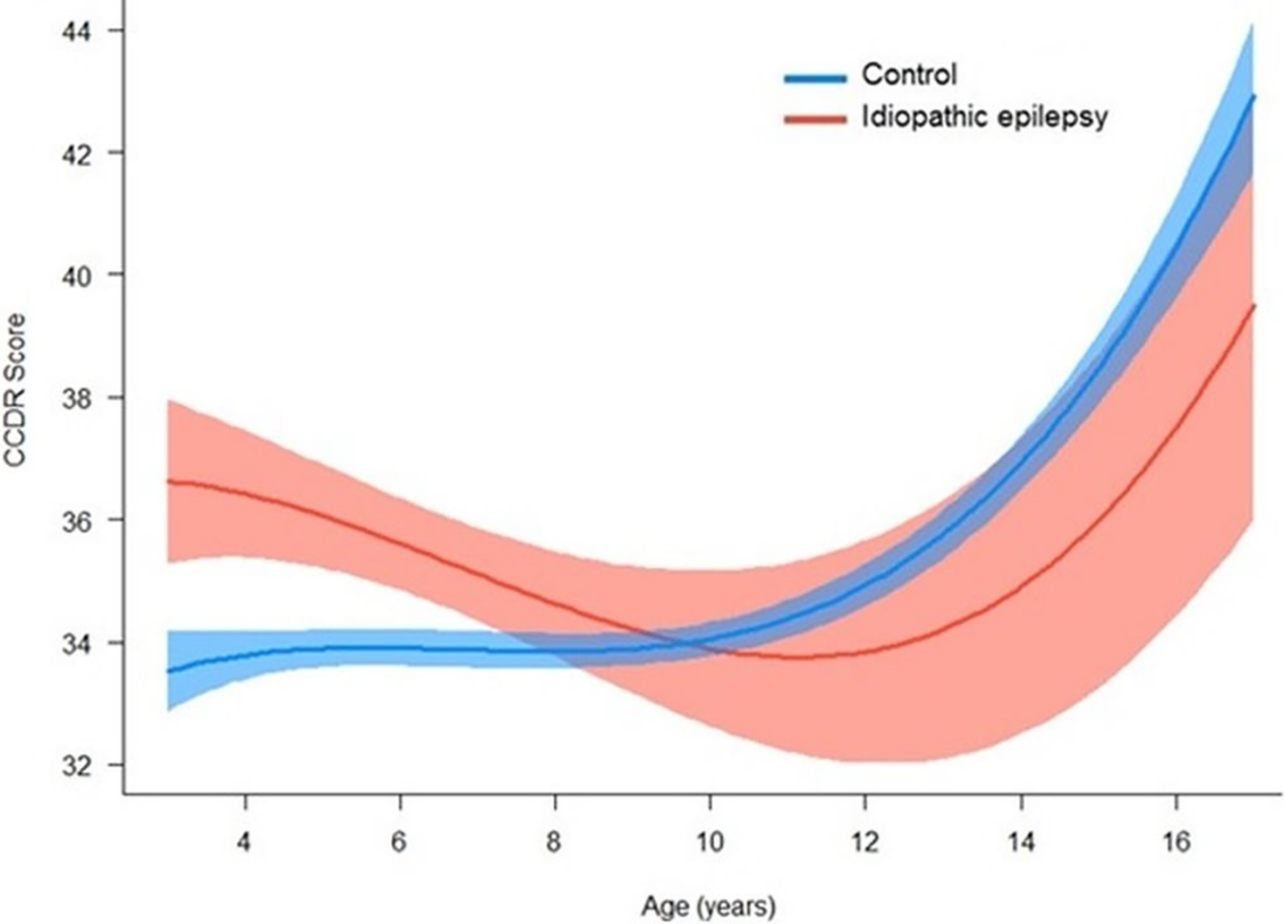
Effect of idiopathic epilepsy on CCDR score across age (years) in dogs with idiopathic epilepsy (n = 286) and controls (n = 3765).

In adult dogs <8 years, dogs with IE had significantly higher CCDR scores which steadily declined with age, before increasing in the geriatric period. In contrast, control dogs CCDR scores remained relatively steady until ~10 years, after which CCDR scores increased exponentially (Fig 4).

### Nature of cognitive impairment

Eleven of the thirteen median scores for the subcomponents of the CCDR scale significantly differed between epilepsy and control dogs. Of the seven ‘static’ “how often does your dog” questions, 6/7 scores were significantly higher (i.e. abnormalities were seen more frequently) in the IE group (Table 7). Of the six ‘progression’ questions, where current behaviour was compared to six months previously, 5/6 differed between groups. Time spent active compared to six months ago was significantly less in dogs with IE than controls. However, four progression scores were significantly higher in the control group; how often they stared blankly at the walls or floor, how often the dog was reported to urinate or defecate in an area it has previously kept clean, how often the dog had difficulty finding food dropped on the floor, and how often they failed to recognise familiar people or pets.

**Table 7:** Differences in the 13 sub-questions of the canine cognitive dysfunction rating (CCDR) scale between dogs with epilepsy (IE) and controls (C).

**Static question**	**Factor**	**Group**	**Score**		**Linear regression**				
			**Mean**	**SD**	**B (IE)**	**P (IE)**	**B (Age)**		**P (Age)**
How often does your dog?	Pace up and down, walk in circles and/or wander with no direction or purpose?	IE	1.85	1.29	0.61	**<0.001**	0.04		**<0.001**
		C	1.31	0.93					
	Stare blankly at the walls or floor?	IE	1.62	1.13	0.48	**<0.001**	0.04		**<0.001**
		C	1.20	0.72					
	Get stuck behind objects and is unable to get around?	IE	1.33	0.87	0.27	**<0.001**	0.02		**<0.001**
		C	1.10	0.49					
	Fail to recognise familiar people or pets?	IE	1.17	0.56	0.13	**<0.001**	0.01		**<0.001**
		C	1.07	0.40					
	Walk into walls or doors?	IE	1.47	0.97	0.41	**<0.001**	0.02		**<0.001**
		C	1.10	0.48					
	Walk away while, or avoid, being patted?	IE	1.47	1.01	0.05	0.426	-0.01		0.089
		C	1.41	0.94					
	**Factor**	**Group**	**Mean**	**SD**					
	Have difficulty finding food dropped on the floor?	IE	1.32	0.65	0.17	**<0.001**	0.03		**<0.001**
		C	1.21	0.52					
**Progression question**	**Factor**	**Group**	**Mean**	**SD**					
Compared with six months ago, does your dog?	Pace up and down, walk in circles and/or wander with no direction or purpose?	IE	2.78	0.88	-0.05	0.258	0.02		**<0.001**
		C	2.86	0.64					
	Stare blankly at the walls or floor?	IE	2.74	0.93	-0.09	**0.022**	0.01		**0.002**
		C	2.85	0.62					
	Urinate or defecate in an area it has previously kept clean?	IE	2.64	0.94	-0.10	**0.028**	0.02		**<0.001**
		C	2.89	6.86					
	Have difficulty finding food dropped on the floor?	IE	5.35	1.73	-0.17	**<0.001**	0.01		**<0.001**
		C	5.74	1.22					
	Fail to recognise familiar people or pets?	IE	7.90	2.60	-0.20	**<0.001**	0.003		0.316
		C	8.52	1.78					
	**Factor**	**Group**	**Mean**	**SD**					
	Is the amount of time your dog spends active?	IE	3.31	0.89	0.27	**<0.001**	0.07		**<0.001**
		C	3.17	0.73					

### Epilepsy sub-population

The mean CCDR score for dogs with IE was 35.05 ± 8.83, with 11 of the 286 dogs CCD+ (3.8%) having reached the threshold score for diagnosis of CCDR ≥50. Of the 286 dogs meeting the criteria for IE diagnosis, the majority (70.3%) were purebred, with the most common breeds being the Border Collie (n = 34, 11.9%) and Labrador Retriever (n = 16, 5.6%). The median age was 6.12 years (4.65–8.04) and weight was 22.0 kg (14.0–34.0). Nearly two-thirds of dogs were male (n = 187, 65.4%) and most were neutered (n = 248, 86.7%). There was no effect of any of these variables on CCDR score (p>0.05). The median age at first seizure was 32.0 months (18.0–48.50), with the median duration of epilepsy being 33.0 months (19.0–56.0). Most dogs (75.5%, n = 216) had experienced cluster seizures, and 40.9% (n = 117) were reported by owners to have entered status epilepticus. The median number of seizures/month (calculated over the preceding three months) was 1.0 (0.0–1.67). Reports of cluster seizures, status epilepticus and higher seizure frequency were associated with a higher CCDR score (Table 8).

**Table 8:** Univariate associations between epilepsy phenotype and canine cognitive dysfunction (CCD) score in the sub-group of dogs with IE.

Variable	Category	N	CCDR Score	B	95% CI	p
Age at first seizure	-	269	-	0.01	-0.05–0.06	0.767
Duration of epilepsy	-	269	-	-0.02	-0.05–0.01	0.248
Seizure frequency	-	276	-	0.547	0.170–0.925	**0.005**
Cluster seizures	No clusters	65	32.18 ± 1.04	-3.81	-6.23 - -1.40	**0.002**
	Clusters	216	36.00 ± 8.74	-		
Status epilepticus	No	197	34.41 ± 8.26	-2.41	-4.73 - -0.09	**0.042**
	Yes	77	36.82 ± 9.98	-		

### Anti-epileptic drugs

Nearly all IE dogs were receiving anti-epileptic medication at the time of reporting (88.8%, n = 254). The most common AEDs used were phenobarbital (63.3%, n = 181), followed by potassium bromide (KBr: 30.4%, n = 87), levetiracetam (LEV: 25.9%, n = 74), imepitoin (IMP: 15.7%, n = 45) and zonisamide (ZNS: 10.8%, n = 31). Dogs treated with LEV had a significantly higher CCDR score than those not treated with this AED, but there were no effects of other AEDs use, or of the number of AEDs used (Table 8). One fifth of dogs had been seizure-free since the commencement of their most recent AED regime (19.8%, n = 53). Nearly one quarter of dogs had had no change in seizure frequency (23.6%, n = 57), while 8.7% (n = 21) had had an increase in seizure frequency. Dogs who had undergone an increase in seizure frequency following their most recent AED regime implementation had higher CCDR scores than seizure-free dogs (Table 9).

**Table 9:** Univariate associations between anti-epileptic drug treatment and canine cognitive dysfunction (CCD) score (from 16–80) in the sub-group of dogs (n = 286) with epilepsy. SF = Seizure frequency.

Variable	Category	N	CCDR Score	B	95% CI	p
Number of AEDs	None	32	34.41 ± 6.85	*Reference*		
	1	128	34.46 ± 8.26	0.06	-3.38–3.49	0.975
	2	38	34.92 ± 10.71	0.51	-3.12–4.13	0.782
	3	37	37.73 ± 8.08	3.32	-0.87–7.52	0.120
	4	6	36. 33 ± 2.07	1.93	-5.80–9.66	0.624
Imepitoin	Yes	45	34.65 ± 9.67	-0.96	-3.78–1.87	0.507
	No	241	35.20 ± 86.65	*Reference*		
Phenobarbital	Yes	181	35.56 ± 9.34	1.39	-0.74–3.52	0.201
	No	105	34.34 ± 7.71	*Reference*		
Potassium Bromide	No	87	35.59 ± 8.40	0.77	-1.47–3.01	0.497
	Yes	199	34.91 ± 8.97	*Reference*		
Levetiracetam	Yes	74	36.82 ± 9.29	2.40	0.06–4.73	**0.044**
	No	212	34.51 ± 8.55	*Reference*		
AED response	Increased SF	21	37.71 ± 9.12	4.49	0.12–8.86	**0.044**
	<50% reduction SF	10	38.90 ± 7.25	5.67	-0.17–11.52	0.057
	No change SF	57	34.86 ± 8.12	1.63	-1.60–4.87	0.321
	>50% reduction SF	101	35.39 ± 8.96	2.16	-0.72–5.04	0.140
	Seizure free	53	33.23 ± 8.45	*Reference*		

In a multivariate analysis, two factors significantly predicted CCDR score (Table 10); higher seizure frequency (quadratic) and history of cluster seizures.

**Table 10:** Generalised linear model of risk factors for CCDR score in the sub-population of dogs with epilepsy (n = 286).

Variable	Category	N	Estimate	SE	t	p
Intercept	-	-	31.54	1.09	29.97	**<0.001**
Seizure frequency	-	276	1.14	0.39	2.90	**0.004**
Seizure frequency ^ 2 (quadratic)	-	276	-0.03	0.02	-2.02	**0.044**
Cluster seizures	No clusters	65	2.74	1.27	2.16	**0.032**
	Clusters	216	Reference			

## Discussion

This study is the first to explore the interplay between epilepsy and cognitive dysfunction in the domestic dog, and is the largest epidemiological study of canine cognitive brain ageing to date. The tool used to quantify cognitive dysfunction in this study, the CCDR, is psychometrically validated and focuses on multiple areas of cognition that are compromised in CCD, and resemble many of the different stages of human dementia [34]. A significantly increased risk of CCD was observed in dogs with IE compared to control dogs. However, based on the current data, it appears likely that epilepsy-associated cognitive dysfunction represents a different form of cognitive impairment than the classic ‘canine dementia’ of CCD. This finding is based on several factors including the (i) nature, (ii) progression and (iii) age of onset of the impairment, which will now be discussed.

### Nature and progression of cognitive impairment in canine epilepsy

When considering specific areas of cognitive compromise captured by the CCDR, dogs with IE showed significantly greater impairment in six of the seven ‘static’ elements, which chiefly reflect impairment in memory. These elements include reduced of recognition of owners, and impairment in spatial memory/orientation, including staring blankly, getting lost in the home, and having difficulty finding food. Compromised memory is common in people with epilepsy, and is often seen early in the course of epilepsy, including childhood epilepsies. In a review of 88 studies of memory in children with epilepsy, 78% of the studies reviewed reported that children with epilepsy scored lower than controls and normative scores on measures of memory [42]. The severity of memory impairments in people with epilepsy may be comparable to patients with precursors to AD [43], and thus the use of the CCDR may lead to false positives in dogs with epilepsy. In contrast to memory function, dogs with IE did not appear to show impairment in social interaction, rarely walking away while, or avoiding, being patted, with no difference in the frequency of these behaviours compared to control dogs. Alterations in social interactions have been considered a core sign of CCD [44], thus adding to the argument that cognitive impairment in IE differs in aetiology from that of geriatric-onset CCD

With the exception of the amount of time spent physically active, dogs with IE did not show greater ‘progression’ of CCD signs than controls. Indeed, control dogs showed significantly worse impairment in four of the five progression elements of the CCDR, which compare behaviour to six months previously. Progressive deterioration in CCD signs in control dogs likely reflects the cognitive ageing process, with cognitive and neurobiological changes observed in dogs entering middle age, and becoming more pronounced as they become geriatric [45]. In contrast, the lack of progression in dogs with IE may reflect their cognitive impairment progressing at an earlier stage than when owners were surveyed, and thus CCD signs have stabilised at the point of this cross-sectional study. Longitudinal study of dogs with IE from age at onset are required to explore this effect further.

### Age of onset of cognitive impairment in canine epilepsy

In addition to the static nature of cognitive compromise in dogs with IE, a significant interaction between epilepsy and age was identified, such that dogs with IE exhibited significantly higher CCDR scores at a young age (<4 years). This is in contrast with control dogs, for whom CCD risk remained extremely low while young-middle aged, and did not elevate until dogs were in the geriatric phase (>10 years), the age at which CCD is most commonly diagnosed. These findings may point towards an initial insult to cognitive function associated with epilepsy, rather than a progressive cognitive deterioration as classically seen in CCD in dogs and dementia in humans. Further data are required to characterise cognitive ageing associated with canine epilepsy. The lower limit for a diagnosis of IE is generally considered to be six months [38], but may be as high as six years. As this study focused on adult dogs, those <3 years of age were not included. Future studies that assess cognition from the onset of epilepsy will help to characterise initial deficits.

Although from the current statistical models CCD risk reduces with age in dogs with IE, there was a paucity of CCDR data for dogs with epilepsy aged >10 years old. The low numbers in this sub-population limit our interpretation of the statistical models that compare geriatric dogs with epilepsy *vs*. controls in the age bracket for which CCD prevalence peaks in the control population. The relative shortage of dogs in this age group may well reflect the reduced longevity of dogs with epilepsy. The median longevity in a study of 102,609 dogs attending UK first opinion veterinary practices was 12.0 years (interquartile range: 8.9–14.2) [46]. In contrast, in longevity studies of canine epilepsy populations, the estimated median age at death was markedly lower, estimated at 7 years of age in two separate studies (median: 7.0, interquartile range: 3.0–10.0; [47]; median: 7.0, interquartile range: 3.0–11.0; [48]). Indeed, the median survival time after the first seizure has been estimated to range from only 2.07 years [49] to 2.3 years [48]. Several factors predict reduced survival time in dogs with epilepsy, including poor seizure control and a high initial seizure frequency [50]. As increased seizure frequency was associated with higher CCDR score in the epilepsy sub-population, it is possible that the prevalence of CCD in that population may be underestimated, if those dogs were at an elevated risk of premature death and thus censored from this study.

### Epilepsy-induced impairment vs. canine dementia

The current data demonstrate similarities in some elements of cognitive impairment in dogs with IE and dogs with geriatric-onset cognitive dysfunction, for example, memory function is impaired in both. However, the progression and age of onset of cognitive dysfunction observed in the IE population appears to diverge from that seen in CCD, which likely points to divergent aetiology. Rather than the pathophysiological mechanisms underlying age-related brain changes and dementia, it is possible that epilepsy interferes with the development of the canine brain networks which underlie cognition [16]. This aetiology may simultaneously contribute to the generation of seizures and to lasting changes in neural circuitry that compromise the canine brains’ ability to process and store information, reflected in memory impairments. These impairments may result from temporary disruption of neural activity patterns, and become permanent if seizures are more frequent [42]. As seen within the current IE sub-population (n = 286), an elevated risk of cognitive dysfunction was observed in those dogs with the most seizure activity (cluster seizure activity and higher seizure frequencies). Seizure frequency has been associated with the degree of mental decline in humans [51], and memory is considered the cognitive function most vulnerable to seizures [52]. From the current cross-sectional data, it remains unclear whether this effect in dogs is a reflection of (i) an underlying epilepsy phenotype characterised by degenerative cerebral processes that give rise to cognitive impairment and high seizure frequency, or (ii) progressive damage from recurrent seizures. Longitudinal studies, including cognitive testing combined with neuroimaging (e.g. hippocampal volume), could provide evidence to test either hypothesis.

### Impact of anti-epileptic drug treatment

In the current multivariate analysis, there was no effect of AED treatment on CCDR score, which is surprising given that AEDs are a major contributor to cognitive impairment in people with epilepsy [53]. This finding should be taken with caution, as 88% of the epilepsy population studied was being treated with AEDs, and thus the control population was of only limited size. AED-induced adverse cognitive and psychotropic effects are thought to reflect suppression of neuronal excitability or enhancing inhibitory neurotransmission, but the actual neurobiological mechanisms of AED-induced cognitive dysfunction are poorly understood [54]. To date, the potential cognitive side-effects of AEDs have been largely neglected in veterinary science, and thus few data on these effects exist.

### Risk factors for CCD

The current study identified age as a major predictor of CCD risk, as has been reported in previous studies [31–33]. In control dogs, CCD prevalence was observed to increase with age, as has been previously documented, with peak prevalence observed in dogs aged >14 years old in both this study and a previous cross-sectional study [33]. Lighter dogs were found to be at an increased risk of CCD in this study, when accounting for age. In a previous study, small dogs (<15kg) had a significantly greater prevalence of CCD than medium-large dogs (>15kg) [32]. In contrast, two previous studies did not find an effect of size on CCD risk, when measured by height at the withers [33] and body weight [31]. This finding may be unexpected, as it is well established that larger dogs size die at a younger age [55], which is presumed to be as a function of more rapid aging of the body. This hypothesis would predict the opposite findings, that larger dogs have an increased risk of CCD than small dogs [56]. However, no studies to date have identified this effect, and from these findings it appears that processes in the brain responsible for age-related behavioural changes may progress more rapidly in smaller dogs.

Finally, dogs with a higher training history score were found to be at lower risk of CCD, which may indicate neuroprotective effects of life experiences that enhance cognition. Higher levels of training have been found to enhance peformance in dogs in various problem-solving tasks [57–59]. Previous studies have found that lifelong training can have protective effects on cognitive function in dogs, for example, increased levels of lifelong training seem to delay or reduce the aging of attentiveness, a cognitive function that declines with age [60]. The possibility of a connection between life experience and the prevalence of dementia has long been discussed in human medicine, with the concept of “cognitive reserve” (CR) increasingly being investigated to account for individual differences in susceptibility to dementia or AD-related pathology [61]. The concept of reserve accounts for individual differences in susceptibility to age-related brain changes or Alzheimer's disease-related pathology. There is evidence that some people can tolerate more of these changes than others and still maintain function. Epidemiologic studies suggest that lifetime exposures including educational and occupational attainment, and leisure activities in late life, can increase this reserve. For example, there is a reduced risk of developing Alzheimer's disease in individuals with higher educational or occupational attainment. It is convenient to think of two types of reserve: brain reserve, which refers to actual differences in the brain itself that may increase tolerance of pathology, and cognitive reserve. Cognitive reserve refers to individual differences in how tasks are performed that may allow some people to be more resilient than others. The concept of cognitive reserve holds out the promise of interventions that could slow cognitive aging or reduce the risk of dementia.Epidemiological studies suggest that lifetime exposures including educational and occupational attainment [62], and leisure activities in later life [63], can increase this reserve, resulting in a reduced risk of developing dementia or AD. Based on the results of 22 cohort studies that examined the effects of education, occupation, premorbid IQ and mental activities on dementia risk, higher CR decreases the risk of developing dementia by 46% [64]. The reduced lifespan of dogs in comparison to humans may allow for prospective intervention studies to initially confirm this effect, and to further identify which activities e.g. obedience or agility training, may protect against CCD in later life.

## Conclusions

This study provides initial evidence of increased levels of cognitive dysfunction in dogs with idiopathic epilepsy. Although a screening tool for CCD (‘canine dementia’) was used to quantify cognitive abnormalities, the age of onset, nature and progression of cognitive impairment in dogs with epilepsy appears divergent from those seen in CCD. Other pathological processes are likely to underlie these abnormalities. Further longitudinal studies measuring cognition from the time of seizure onset are required to further characterise the progression of cognitive dysfunction in this population, with additional data (where possible) required from geriatric dogs with epilepsy to explore the interplay between epilepsy-induced cognitive impairment, and impairments related to normal ageing and CCD. In addition, clinicopathological studies are needed in dogs with IE to determine if, like in human epilepsy, they also express higher levels of Alzheimer pathology than normal. This study also identified age, size and training history as risk factors for CCD. Positive effects of training experiences should be further explored in the canine population, to identify whether, as in humans, they can increase cognitive reserve and protect against CCD.

## Supporting information

S1 Dataset (XLSX)

S1 File The Mature Dog Survey. Online survey questions including the canine cognitive dysfunction rating scale (CCDR).
(PDF)

## Abbreviations

AβB: β-amyloid
AD: Alzheimer’s disease
AED: Anti-Epileptic Drug
AIC: Akaike information criterion
CCD: Canine cognitive dysfunction
CCDR: Canine cognitive dysfunction rating scale
CR: Cognitive reserve
IE: Idiopathic Epilepsy
IVETF: International Veterinary Epilepsy Task Force
IQ: Intelligence Quotient
TLE: Temporal Lobe Epilepsy
MRI: Magnetic Resonance Imaging
SF: Seizure frequency
QoL: Quality of Life
