# Supplementary material for: Cognitive dysfunction in naturally occurring canine idiopathic epilepsy
Source: PLoS One. 2018 Feb 8;13(2):e0192182. doi: 10.1371/journal.pone.0192182 (PMC5805257; doi:10.1371/journal.pone.0192182)
Supplement: S1 File — Online survey questions including the canine cognitive dysfunction rating scale (CCDR). (PDF) [file pone.0192182.s002.pdf]

Royal Veterinary College
University of London

Mature Dog Survey

Introduction

What is this survey about?

We are interested in the impact of your dogs' health and training upon their behaviour. This survey will explore your dog's current health status (with different questions depending on current disorders), day-to-day behaviour and training.

We are interested in both dogs affected by health conditions, and healthy dogs with no diagnosed health conditions. We invite owners of dogs aged over 3 years old, of all breeds and cross-breeds to complete this survey.

We would be grateful if you could complete the questionnaire only once for each dog in your care. For your answers to be of use you have to complete the full questionnaire, which is estimated to take 20 minutes. We very much appreciate your help in this study. If you have any queries regarding the questions asked, please contact Dr Rowena Packer (rpacker@rvc.ac.uk)

Data Protection and Consent

The data collected in this questionnaire will be collated and stored at the Royal Veterinary College in London (RVC). Your completion of the questionnaire indicates your consent to participate in this study. We would appreciate it if you could include your contact details, but this is not required if you prefer your answers to remain anonymous. Your data will be anonymised as appropriate, and your contact details will be used only to contact you if we need clarification of the answers to any questions forming part of this questionnaire. Your personal information will be held and used in accordance with the Data Protection Act 1998 and will not be disclosed to any unauthorised person or body. We are happy to send you an electronic summary of the study results (by email only) at the end of the study. Only the principal investigators in this study (Prof Holger Volk, Dr Rowena Packer, Prof Paul McGreevy) and final year veterinary students working on this project (Ms Amy Pergande and Ms Chloe Chaplin) will have access to the data on the returned questionnaires. The anonymous results will be published on the RVC website and in a journal.

N.B. The study was approved by the Royal Veterinary College Ethics and Welfare Committee (2016/U301 and 2016/U129)

Royal Veterinary College
University of London

Mature Dog Survey

About You

1. Date of completion of the questionnaire

	MM	DD	YYYY
Date	/	/	

2. In which country do you and your dog reside?

3. Where did you hear about this questionnaire?

- Your normal veterinary practice
- Your referral/specialist vet
- Facebook page
- Twitter

Other (please specify)

* 4. Please select your gender

- Male
- Female
- Prefer not to say

* 5. Please select your age range

- ◯ 18-30
- ◯ 31-45
- ◯ 46-60
- ◯ 61-75
- ◯ over 76
- ◯ Prefer not to say

Royal Veterinary College
University of London

Mature Dog Survey

Your dog

* 6. Is your dog pure-bred or cross-bred?

- ◯ Pure-bred
- ◯ Cross-bred

Royal Veterinary College
University of London

Mature Dog Survey

Your dog (continued)

7. If your dog is pure-bred, is your dog registered with a breed association or kennel club in your country?

- Yes
- No
- I don't know
- Other (please specify)

* 8. What breed is your dog?

If your breed is not listed please state here. If your dog is cross-bred then please state the breeds it is bred from here, if known.

* 9. What sex is your dog

- Female entire
- Female neutered
- Male entire
- Male neutered

10. If your dog is neutered, at what age was it neutered?

* 11. How old is your dog? (N.B. If your dog is less than 3 years old, please do not complete this survey)

12. How old was your dog when you bought/rehomed them?

13. What is your dogs weight in kilograms (to the nearest kg)?

* 14. Has your dog had any of the following health problems diagnosed by a vet? (tick all that apply)

	No (Unaffected)	Yes (Mildly affected)	Yes (Moderately affected)	Yes (Severely affected)
Ear canal disease (otitis externa)				
Dental disease (peridontal disease)				
Anal sac impaction				
Overgrown nails				
Arthritis (degenerative joint disease)				
Gastrointestinal disease (Diarrhoea/Vomiting)				
Obesity				
Injuries (traumatic injuries)				
Conjunctivitis				
Heart murmur				
Canine cognitive dysfunction				

Other (please explain):

15. Is your dog currently on any prescribed medication (other than routine worming/vaccinations, or anti-epileptic drugs used to control your dog's epilepsy, if your dog is affected by epilepsy)?

- ◯ Yes
- ◯ No

If yes, what medications? (please list brand names where possible)

16. Does your dog have health insurance?

- ◯ Yes
- ◯ No
- ◯ I don't know

Royal Veterinary College
University of London

Mature Dog Survey

Veterinary history

* 17. Has your dog ever had a seizure?

- ○ Yes
- ○ No
- ○ I don't know

* 18. Has your dog been diagnosed with epilepsy by a vet?

- ○ Yes
- ○ No

Royal Veterinary College
University of London

Mature Dog Survey

Epilepsy diagnosis

* 19. To your knowledge, has your dog had 2 or more seizures (if only 2, they were at least 24 hours apart)?

- ◯ Yes
- ◯ No

* 20. Was your dog's first seizure between the ages of 6 months and 6 years?

- ◯ Yes
- ◯ No

* 21. Has a vet carried out blood and urine tests on your dog and found no identifiable cause for his/her seizures?

- ◯ Yes
- ◯ No

Royal Veterinary College
University of London

Mature Dog Survey

Your dog's epilepsy

* 22. How old was your dog when the first seizure occurred? (If not known, select 'Unknown')

23. How many fits has your dog had in total? (if a precise number has not been recorded, please estimate to the nearest 10). If completely unknown, select 'Unknown'

24. Which vet did you see to discuss your dog's seizures?

- ○ My local vet(s) only
- ○ My local vet(s) AND a Neurology Specialist

* 25. Which of the following tests were carried out by your vet(s) to diagnose epilepsy? Tick all that apply

- ☐ Blood tests
- ☐ Urine tests
- ☐ MRI scan of the brain
- ☐ CT scan of the brain
- ☐ Lumbar puncture/ Cerebrospinal fluid (CSF) analysis
- ☐ Tests were done, but I am uncertain of the details

* 26. What was the diagnosis for your dog's seizures?

- ○ Idiopathic epilepsy (i.e. no cause found)
- ○ Other causes e.g. a brain tumour, liver problems, a stroke, meningitis

27. When did your dog last have a seizure?

- ◯ Within the last 24 hours
- ◯ Within the last 7 days
- ◯ 8-14 days ago
- ◯ 15-21 days ago
- ◯ 22-28 days ago
- ◯ 1-3 months ago
- ◯ 4-6 months ago
- ◯ 7-12 months ago
- ◯ Over 12 months ago
- ◯ I don't know

28. Approximately how many seizures has your dog had in the past three months?

[ ]

* 29. Has your dog ever had more than one seizure in one day (24 hour period) - also known as a 'cluster seizure'?

- ◯ No - only one fit has ever occurred within 24 hours
- ◯ Yes - more than one fit has occurred within 24 hours
- ◯ I don't know

* 30. Has your dog ever experienced a seizure that lasted for longer than 5 minutes?

- ◯ Yes
- ◯ No
- ◯ I don't know

* 31. Has your dog ever had two or more seizures in a row without fully regaining consciousness in between them?

- ◯ Yes
- ◯ No
- ◯ I don't know

32. Does your dog currently receive any medication to treat their epilepsy?

- ◯ Yes
- ◯ No

Royal Veterinary College
University of London

Mature Dog Survey

Epilepsy treatment

* 33. Does your dog CURRENTLY receive any of the following epilepsy medications? (Tick as many as apply)

- [ ] Imepitoin (Pexion)
- [ ] Phenobarbital (Epiphen, Phenoleptil)
- [ ] Potassium Bromide (Epilease, Libromide)
- [ ] Levetiracetam (Keppra)
- [ ] Zonisamide
- [ ] Gabapentin
- [ ] Diazepam
- [ ] Pregablin
- [ ] Chlorazepate
- [ ] Felbamate
- [ ] Phenytoin
- [ ] Lamotrigine
- [ ] Tiagabine
- [ ] Vigabatrin

34. What doses of medication is your dog currently receiving to control the seizures?

Please copy the information written on the bottles for all medication including medication name, medication strength, dose and frequency

EXAMPLE
Medication: Epiphen, 60mg tablet, 2 tablets, twice daily

Medication ______

Medication ______

Medication ______

Medication ______

Medication ______

Medication ______

35. When did your dog's current epilepsy medication regime commence (N.B. If your dog is on ONE medication, the date this was started. If your dog is on MORE THAN ONE medication, the date that their <u>most recent</u> medication was added)

	DD		MM		YYYY
Date		/		/	

36. How many seizures per month (on average) did your dog have BEFORE this date?

37. How many seizures per month (on average) has your dog had SINCE this date?

38. Does your dog experience side effects from their anti-epileptic medication, and if so, how severe are they? If your dog did not show this side effect, please tick 'not present'. Please tick ALL that apply.

	Not present	Very Mild	Mild	A moderate amount	Severe	Very Severe
Eating more / would like to eat more	○	○	○	○	○	○
Gaining weight	○	○	○	○	○	○
Drinking more	○	○	○	○	○	○
Urinating more	○	○	○	○	○	○
Sleeping more than before	○	○	○	○	○	○
Wobbly / not coordinated when walking	○	○	○	○	○	○
Restlessness / pacing	○	○	○	○	○	○
Itchiness or skin rash	○	○	○	○	○	○
Vomiting	○	○	○	○	○	○
Diarrhoea	○	○	○	○	○	○
Coughing	○	○	○	○	○	○

Royal Veterinary College
University of London

Mature Dog Survey

Your dog's seizures

39. Think about the most common type of seizure your dog has. Which signs does your dog show during this type of seizure? Select ALL that apply

- [ ] Falls on the floor
- [ ] Body goes floppy/limp
- [ ] Body goes stiff
- [ ] Running/paddling movements
- [ ] Facial twitches
- [ ] Chewing movements
- [ ] Rhythmic blinking
- [ ] Head shaking
- [ ] Twitching of one leg
- [ ] Excessive salivation/drooling
- [ ] Vomits
- [ ] Urinates
- [ ] Defecates
- [ ] Pupils dilate
- [ ] Fearful/anxious behaviour
- [ ] Fly snapping
- [ ] Doesn't respond to my voice
- [ ] Cannot look me in the eye
- [ ] Other (please specify)

40. How long do your dog's most common type of seizures last on average? (in minutes)

41. On a scale of 1 (very mild) to 7 (very severe), please rate how severe your dog's most common type of seizures are on average

	1 - Not at all severe	2 - Very mild	3 - Mild	4 - Moderate	5 - Severe	6 - Very Severe	7 - Could not be worse	N/A
Severity	○	○	○	○	○	○	○	○

42. What signs does your dog show while recovering from their <u>most common</u> type of seizure? Select ALL that apply. If your dog is normal immediately after a seizure select 'None of the above'

My dog is:

- ☐ wobbly/ ataxic
- ☐ disorientated
- ☐ staring
- ☐ blind /can't see very well
- ☐ sleepy
- ☐ fearful /scared
- ☐ aggressive
- ☐ restless /pacing
- ☐ sniffing
- ☐ hungry
- ☐ thirsty
- ☐ clingy/wants more attention than normal
- ☐ Normal - none of the above

43. How long does it take for your dog to COMPLETELY recover from their <u>most common</u> seizure type?

My dog:

- ○ is usually completely back to normal within minutes after the fit
- ○ is usually completely back to normal within 1-6 hours after the fit
- ○ is usually completely back to normal within 6 hours after the fi
- ○ is usually completely back to normal within 24 hours after the fit
- ○ is usually completely back to normal within 48 hours after the fit
- ○ usually takes more than 48 hours to recover completely

Royal Veterinary College
University of London

Mature Dog Survey

Your dog's behaviour

* 44. How often does your dog show the following behaviours?

	Never	Once a month	Once a week	Once a day	More than once a day
How often does your dog pace up and down, walk in circles and/or wander with no direction or purpose?	○	○	○	○	○
How often does your dog stare blankly at the walls or floor?	○	○	○	○	○
How often does your dog get stuck behind objects and is unable to get around?	○	○	○	○	○
How often does your pet fail to recognise familiar people or pets?	○	○	○	○	○
How often does your dog walk into walls or doors?	○	○	○	○	○
How often does your dog walk away while, or avoid, being patted?	○	○	○	○	○

* 45. How often does your dog show the following behaviour?

	Never	1-30% of the times	31-60% of the times	61-99% of the times	Always
How often does your dog have difficulty finding food dropped on the floor?	○	○	○	○	○

* 46. Compared with 6 months ago, does your dog show the following behaviours?

	Much less	Slightly less	The same	Slightly more	Much more
Compared with 6 months ago, does your dog now pace up and down, walk in circles and/or wander with no direction or purpose	◯	◯	◯	◯	◯
Compared with 6 months ago, does your dog now stare blankly at the walls or floor	◯	◯	◯	◯	◯
Compared with 6 months ago, does your dog urinate or defecate in an area previously kept clean (if your dog has never house-soiled, tick 'the same')	◯	◯	◯	◯	◯
Compared with 6 months ago, does your dog have difficulty finding food dropped on the floor	◯	◯	◯	◯	◯
Compared with 6 months ago, does your dog fail to recognise familiar people or pets	◯	◯	◯	◯	◯

* 47. Compared with 6 months ago, does your dog show the following behaviours?

	Much more	Slightly more	The same	Slightly less	Much less
Compared with 6 months ago, is the amount of times your dog spends active	◯	◯	◯	◯	◯

Royal Veterinary College
University of London

Mature Dog Survey

Your dog's behaviour

* 48. How often does your dog do the following?

	Never	Seldom	Sometimes	Usually	Always
Returns immediately when called while off leash	○	○	○	○	○
Obeys a sit command immediately	○	○	○	○	○
Obeys a stay command immediately	○	○	○	○	○
Will fetch or attempt to fetch sticks, balls and other objects	○	○	○	○	○
Seems to attend to or listen closely to everything you say or does	○	○	○	○	○
Is slow to respond to correction or punishment	○	○	○	○	○
Is slow to learn new tricks or tasks	○	○	○	○	○
Is easily distracted by interesting sights, sounds or smells	○	○	○	○	○

Royal Veterinary College
University of London

Mature Dog Survey

Training history

* 49. How many puppy classes did your dog attend?

* 50. Apart from puppy classes, has your dog attended obedience training?

- Yes - Attended but has now stopped
- Yes - Still attending
- No- Never attended

51. If your dog attended obedience training but has now stopped, how old were they when they stopped?

* 52. Has your dog taken part in any of the following activities?

	Never	Yes - but has now stopped	Yes - still participates
Agility			
Gundog training			
Ringcraft classes			
Dog showing			
Flyball			

* 53. Which of the following training techniques have you ever used with your dog? (tick all that apply)

- [ ] Food rewards (giving a treat) when the dog does a correct behaviour
- [ ] Bark activated citronella collar (automatically sprays strong smelling liquid to stop barking)
- [ ] Harness to prevent pulling on lead
- [ ] Verbal punishment (e.g. telling off or shouting) when the dog does something wrong
- [ ] Shutting away (physically removing from the room, sometimes called 'time out') when the dog behaves badly
- [ ] Stroking or patting when the dog behaves well, verbal praise
- [ ] Pet corrector (aerosol type spray directed at dog to interrupt unwanted behaviour)
- [ ] Electronic boundary fence to prevent the dog from wandering off the property
- [ ] Physical punishment (e.g. smacking) when the dog does something wrong
- [ ] Withhold treats or food when the dog does something wrong
- [ ] Ignoring (stopping giving the dog any attention when he or she does something wrong)
- [ ] Electronic training collar (to give an electronic correction when the dog does something wrong)
- [ ] Choke chain (metal collar that a tightens on dogs neck) to prevent pulling on lead
- [ ] Playing (e.g. throwing a toy when the dog does a correct behaviour)
- [ ] Physical manipulation (e.g. pushing the bottom down) to encourage correct behaviour
- [ ] Pulling back on lead when the dog pulls
- [ ] Bark activated electronic training collar (automatically gives electronic correction to stop barking)
- [ ] Water pistol (sprayed to interrupt a behaviour when dog does something wrong)
- [ ] "Husher" device that prevents the dog barking
- [ ] Clicker training (using the 'click' sound, followed by a treat when the dog does a correct behaviour)
- [ ] Stopping forward movement or changing direction when the dog pulls on the lead
- [ ] Non-verbal sound distraction (e.g. can of stones, 'training discs', or air horn) to stop the behaviour when the dog does something wrong
- [ ] Prong collar (metal chain with extensions that put pressure on dog's neck when it pulls on the lead)
- [ ] Citronella collar (to give remotely initiated unpleasant smelling spray when the dog does something wrong)
- [ ] Other (please describe):

* 54. How much exercise (in minutes) does your dog receive on an average weekday or weekend, and how much (0-100%) is on lead or off lead?

	Duration	On lead / off lead
Weekday		
Weekend		

Royal Veterinary College University of London

Mature Dog Survey

Thank you and data information

Thank you very much for your time and effort in answering this questionnaire!

Your data will be used only for the current study and the results will be published in a veterinary journal. Summaries of the results will be made available on the RVC website.

55. Occasionally, it may be useful for us to contact you to clarify some of your answers, or to email you the final report on this study. If you consent to being contacted by one of the staff involved in the project, please provide your email address below:
